# Origins of the Resting-State Functional MRI Signal: Potential Limitations of the “Neurocentric” Model

**DOI:** 10.3389/fnins.2019.01136

**Published:** 2019-10-23

**Authors:** Hanbing Lu, Saul Jaime, Yihong Yang

**Affiliations:** ^1^Neuroimaging Research Branch, National Institute on Drug Abuse (NIDA) Intramural Research Program, National Institutes of Health, Bethesda, MD, United States; ^2^Division of Pharmacology & Toxicology, College of Pharmacy, The University of Texas at Austin, Austin, TX, United States; ^3^Waggoner Center for Alcohol & Addiction Research, The University of Texas at Austin, Austin, TX, United States

**Keywords:** resting-state MRI, functional connectivity, MUA, LFP, BOLD

## Abstract

Resting-state functional connectivity (rsFC) is emerging as a research tool for systems and clinical neuroscience. The mechanism underlying resting-state functional MRI (rsfMRI) signal, however, remains incompletely understood. A widely held assumption is that the spontaneous fluctuations in blood oxygenation level-dependent (BOLD) signal reflect ongoing neuronal processes (herein called “neurocentric” model). In support of this model, evidence from human and animal studies collectively reveals that the spatial synchrony of spontaneously occurring electrophysiological signal recapitulates BOLD rsFC networks. Two recent experiments from independent labs designed to specifically examine neuronal origins of rsFC, however, suggest that spontaneously occurring neuronal events, as assessed by multiunit activity or local field potential (LFP), although statistically significant, explain only a small portion (∼10%) of variance in resting-state BOLD fluctuations. These two studies, although each with its own limitations, suggest that the spontaneous fluctuations in rsfMRI, may have complex cellular origins, and the “neurocentric” model may not apply to all brain regions.

## Introduction

Noise exists in all measurements. The significance of spatially coherent “noise” in blood oxygenation level-dependent (BOLD) signal was appreciated in a seminal paper by Biswal et al. (1995), who revealed temporal synchrony in human sensorimotor system. As similar results accumulated for other brain systems (Greicius et al., 2003; Beckmann et al., 2005; Fox et al., 2005; Seeley et al., 2007; Habas et al., 2009; Raichle, 2011), in particular the discovery of the “default mode network (DMN) (Raichle et al., 2001; Greicius et al., 2003),” a concept emerged suggesting that the persistent, correlated spontaneous activity between brain regions [functional connectivity (FC)], initially thought to be noise (i.e., random error) in BOLD measurements, is in fact a meaningful source of information, reflecting a fundamental feature of brain functional organization (Raichle, 2009). Further supporting this view, a recent study defined depression subtypes based on FC patterns; the resulting biotypes predicted individuals’ responsiveness to transcranial magnetic stimulation (TMS) therapy, pointing to clinical potentials of resting-state functional connectivity (rsFC) (Greicius, 2008).

A cornerstone assumption in rsFC is that the ongoing, spontaneously occurring synchrony among brain areas reflects the inherent functional organization of the neural network. The definition of “neural network” in the context of resting-state functional MRI (rsfMRI), however, remains vague. Data accumulated over the past decade, more or less, lend support to the hypothesis that synchronized neuronal activity underlies the BOLD FC. Recent animal studies from two independent labs designed to directly test this hypothesis, however, suggest cautious interpretation of the data (see below). As rsFC becomes increasingly used as a research tool for basic and clinical neuroscience, a thorough understanding of its physiological basis and origin becomes essential and critical.

In the spirit of stimulating scientific debate, in this *opinionated* mini-review, we will start with methodological considerations that we deem important in elucidating the physiological basis of BOLD fluctuations, followed by critical review of recent evidence that supports neuronal origin of the rsfMRI signal, herein we will call it “neurocentric” model; we will then present several lines of recent evidence that appear divergent from this concept. Finally, we will bring forward testable hypotheses from a perspective of BOLD signal transduction. Extensive review of this subject can be found elsewhere (Leopold and Maier, 2012; Lu and Stein, 2013). We will focus on progress in the past 5 years.

## Methodological Considerations

Brain’s electrical signals can be measured at different scales, from intra- and extra-cellular recording, local field potential (LFP) to electroencephalogram and magnetoencephalography (Buzsáki et al., 2012). The LFP signal has been shown to be correlated with BOLD response to a task (e.g., visual stimulation) (Lauritzen, 2001; Logothetis et al., 2001). By extension, LFP is often used to investigate the electrophysiological correlate of the rsfMRI signal (Lu et al., 2007; Mantini et al., 2007; He et al., 2008; Shmuel and Leopold, 2008; de Pasquale et al., 2010; Schölvinck et al., 2010; Pan et al., 2011; Wang et al., 2012; Hutchison et al., 2015; Shi et al., 2019). The underlying assumption is that BOLD fluctuation in the resting-state and the evoked BOLD response to a task manipulation share the same signal transduction mechanism. As will be discussed later in this article, this assumption may require a careful assessment.

On the other hand, BOLD signal results from the mismatch between blood flow and oxygen metabolism (Fox and Raichle, 1986; Kim and Ogawa, 2012), indirectly reflecting neuronal activity. Thus, LFP and BOLD signals are two fundamentally different readouts of brain activity. We have previously argued that (Lu and Stein, 2013), in order for the fMRI signal to be considered as a surrogate of a specific neuronal physiological measure, at a minimum, the following criteria should be met: (i) the temporal fluctuations of the electrical and the BOLD signals should remain correlated, and such correlation should be persistent across brain states; (ii) the spatially correlated patterns from electrical signal should recapitulate that from BOLD signal across brain states; and (iii) each pattern should be unique to each network. To meet the above three criteria, it would appear necessary that both types of signals should be recorded simultaneously on the same subject. Due to substantial technical difficulties and for practical reasons, to the best of our knowledge, most published studies employed experimental designs that partially meet these criteria, and thus should be considered critically.

## Spatially Correlated Patterns of the Electrical Signal Recapitulate Bold rsFC

Perhaps the most intuitive evidence to support the “neurocentric” model is the distinct spatial patterns of the electrical signal, which bear remarkable similarity to BOLD FC (Fukushima et al., 2012; Liu et al., 2015; Hacker et al., 2017; Kucyi et al., 2018; Shi et al., 2019). Data from voltage sensitive dye (VSD) fluorescent imaging appear particularly compelling (Mohajerani et al., 2010). Conventional electrophysiological recording necessitates reference and ground electrodes; and there is more or less “volume conduction effect” (Kajikawa and Schroeder, 2011). Confounds from these technical aspects lead to certain degrees of ambiguity in terms of spatial localization of the electrical signal. This could potentially introduce artifactual inter-regional correlation. VSD imaging measures membrane potential changes, avoiding these confounds entirely.

In a mouse model with a large craniotomy preparation, Mohajerani et al. (2010) simultaneously recorded VSD signals in both hemispheres. Strong oscillations exist in spontaneous ongoing VSD signals, which mirror LFP signal in the low frequency band (3–6 Hz). The oscillations in homotopic cortical regions were correlated; discrete peaks characterized each region. Awake and urethane anesthetized mice showed similar inter-hemispheric synchrony. Furthermore, they found that, in genetically acallosal mice, the interhemispheric synchrony was significantly reduced, a finding similar to rsFC studies in humans (Lowe et al., 1997; Quigley et al., 2003; Johnston et al., 2008). The localized synchrony patterns in homotopic cortical regions recorded using VSD are strikingly similar to rsFC reported in rodent fMRI literature (Lu et al., 2007, 2012; Pawela et al., 2008; Zhao et al., 2008; Hutchison et al., 2010; Liang et al., 2011; Magnuson et al., 2014; Gozzi and Schwarz, 2016), including sensory networks in the forelimb region, whisker cortex, motor cortex etc. Notably, the retrosplenial cortex, a major component of the DMN (Lu et al., 2012), was also depicted in the VSD data.

Spatially correlated patterns between electrophysiological signals recorded in areas of classic FC networks were also observed in humans (He et al., 2008; Hacker et al., 2017; Kucyi et al., 2018). In these studies, patients underwent neurosurgical electrocorticography (ECoG) electrode implantation. Based on clinical needs, each study had a unique cohort of patients with electrodes covering specific brain regions. Taken together, the electrodes covered sensory motor network (SMN), dorsal attention network (DAN), DMN, and frontoparietal control system (FPC). In general, these studies found a higher within network correlation than between network correlation, and there is a spatial correspondence between ECoG and BOLD FC patterns. Less consistent was which frequency band contributes the most to the within and between network correlations. For example, Hacker et al. (2017) showed that the correspondence appeared to be frequency band-specific: theta (4–8 Hz) band-limited power (BLP) correspondence appeared stronger in the DMN and FPC, while theta (8–12 Hz) BLP correspondence was stronger in the SMN and DAN. They also found that gamma BLP correspondence was commonly observed throughout the brain. Kucyi et al. (2018) reported that correlation patterns in high frequency broadband (70–170 Hz) power were consistent during wakeful rest and sleep; although similar correlation pattern exist in lower-frequency (1–70 Hz) power, but the spatial specificity and temporal consistency were inferior to higher frequency broadband power.

A recent study by Shi et al. (2019) recorded ECoG signal from the primary somatosensory cortex (areas 3b and 1) of anesthetized monkeys, and found that spontaneous fluctuations in low frequency LFP signal was the major contributor to resting-state LFP coherence. Furthermore, they reported that the temporal dynamics in BOLD FC behaved most similarly to the low frequency LFP coherence. These results are generally in line with the conclusions from studies in anesthetized rats (Lu et al., 2007, 2014; Pan et al., 2010; Magnuson et al., 2014). It is not clear whether the use of anesthesia in rats and monkeys played a role in the discrepancy mentioned above.

In summary, evidence from animals and humans collectively suggests a high degree of correspondence in spatial correlation patterns derived from electrophysiological recording and BOLD rsFC, it would appear tempting to conclude that the electrophysiological signal underlies BOLD rsFC. However, making this leap requires correspondence in temporal behavior of these two types of brain readouts, which necessitates simultaneous measurement of both types of signals. Unfortunately, two lines of evidence from simultaneously recorded electrophysiological and hemodynamic signals seem difficult to reconcile with the “neurocentric” model.

## Lfp–Bold Correlation is Weak

**FIGURE 1:**
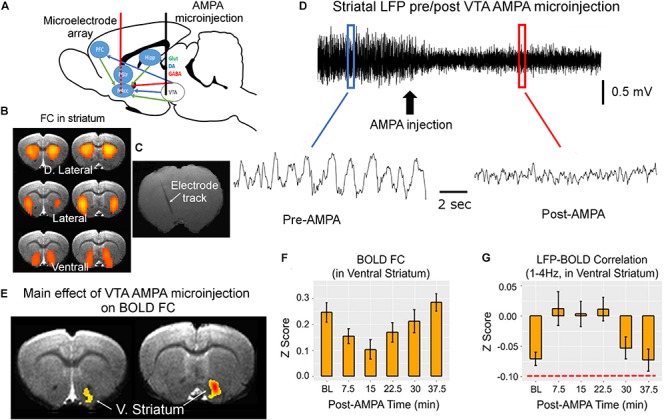
MRI-compatible linear electrode array was implanted into the rat striatum covering the dorsolateral and ventrolateral domains **(A–C)**. These domains were identified based on the three bilateral rsFC network in rat striatum that correspond to the three functional domains of the rat striatum **(B)**. Alpha-amino-3-hydroxy-5-methyl-4-isoxazole propionic acid (AMPA) was injected into the ventral tegmental area (VTA) via a guide cannula to enhance the activity of VTA dopaminergic neurons, which project primarily to the ventral striatum, modulating striatal local field potential (LFP) signal **(D)**. The waveforms before and after AMPA injection were derived from the blue and red boxes, respectively. **(E,F)** The main effect of AMPA on BOLD functional connectivity (FC). **(G)** The effects of VTA AMPA microinjections LFP–BOLD correlation (corr.). Note the low correlation value in LFP–BOLD correlation, indicated by red dashed line in panel **(G)**. All maps are thresholded at *p* < 0.05 after correction for multiple comparisons. DA, dopamine; D. Striatum, dorsal striatum; V. Striatum, ventral striatum (nucleus accumbens); GABA, gamma-aminobutyric acid; Glut, glutamate; Hipp, hippocampus; NAcc, nucleus accumbens core; and PFC, prefrontal cortex (adapted with permission from the authors).

We developed a simultaneous fMRI–electrophysiological recording technique (Jaime et al., 2018), and performed chronic repetitive recordings in rat striatum. The electrophysiological recording and BOLD data acquisition were coupled with pharmacological modulation of the well-defined dopaminergic pathway (Figure 1A). We found three distinct BOLD rsFC networks using the independent component analysis. These three networks are consistent with well-known three functional domains in rat striatum (Voorn et al., 2004). We thus implanted silicon-based microelectrode array (16 contacts) that covered the dorsolateral to ventral medial striatum (Figures 1B,C). With microinjection of alpha-amino-3-hydroxy-5-methyl-4-isoxazole propionic acid (AMPA) receptor agonist into the ventral tegmental area (VTA), we systematically modulated dopamine release and neuronal activity in the striatum, to which VTA dopamine neurons project most densely. As shown in Figure 1D, both the amplitude and the frequency of the striatal LFP signal were modulated. VTA AMPA microinjection significantly modulated FC only in the ventral striatum (nucleus accumbens) (Figures 1E,F), consistent with known neuroanatomy.

Concurrent LFP and BOLD signal recording allowed us to directly interrogate the temporal relationship of these two types of brain readouts. Perhaps the most unexpected finding is the low correlation between LFP and BOLD signal: although the LFP-BOLD time courses were statistically correlated, the average LFP-BOLD correlation was below 0.1 (Figure 1G). The correlation between gamma LFP and the fMRI signal was similar but opposite in sign (Jaime et al., 2017). These data suggest that spontaneous LFP fluctuations explain only a small portion of variance in BOLD fluctuations (Sumiyoshi et al., 2019).

**FIGURE 2:**
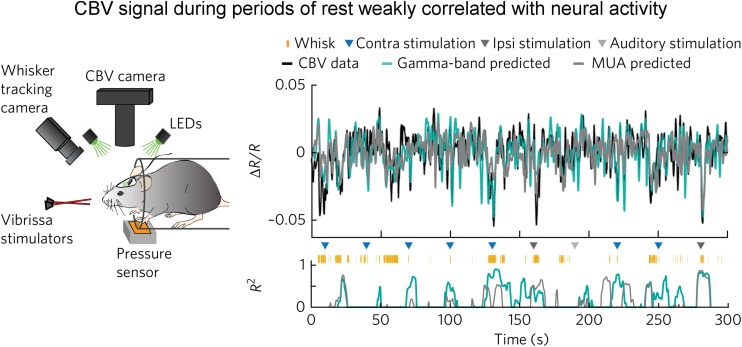
Weak correlation between electrophysiological and hemodynamic signal during periods of “rest.” **(Left)** Schematic of the experimental setup for optical imaging of intrinsic signal (the isosbestic point green light measures total hemoglobin signal, reflecting CBV). Animal’s behavior and movement were dynamically monitored. **(Right)** Predictions of ongoing CBV for a single trial. **(Top)** Predictions by gamma-band (teal blue, *R*^2^ = 0.29) and MUA-derived (gray, *R*^2^ = 0.20) HRFs. **(Bottom)** Goodness-of-fit (*R*^2^) for 8-s sliding windows. Colored triangles indicate sensory stimuli. Orange tick marks indicate volitional whisking events. Note that *R*^2^ was low during the “rest.” Here, the “rest” was defined as the periods absent of experimenter-delivered stimulation, fidgeting, and volitional whisking events (adapted with permission from the authors).

The above observation by Jaime et al. (2017) is corroborated by a recent optical imaging study (Winder et al., 2017). In this study, Winder et al. (2017) applied intrinsic optical imaging to measure hemodynamic signal [total hemoglobin signal, reflecting cerebral blood volume (CBV)] while at the same time recording neuronal activity in the whisker barrel cortex of the awake, head-fixed mice. What made this study especially unique in the context of spontaneous brain activity was that they carefully monitored whisker and body movements. They found that spontaneous CBV changes in the absence of experimenter-delivered sensory input were largely driven by volitional whisker and body movements. During periods of “rest” when there was no experimenter-delivered sensory stimulation, volitional whisker and body movements, CBV signal was only weakly correlated with neural activity assessed with either gamma band LFP or multiunit activity (with an *R*^2^ of about 0.1, Figure 2). They performed pharmacological manipulations to block local neural spiking, glutamatergic input and noradrenergic receptors, and found that spontaneous fluctuations in CBV and vessel diameter persisted, indicating that spontaneous CBV fluctuations may have a non-neuronal origin.

The results reported by Winder et al. (2017) are divergent from several recent optical studies (Matsui et al., 2011; Ma et al., 2016; Mateo et al., 2017). For example, Ma et al. (2016) reported that spontaneous CBV fluctuations highly correlated with ongoing neuronal activity as measured with GCaMP optical imaging. The reasons for this discrepancy are unknown, but one might speculate that the differential definition of “resting-state” may have played a role, an issue particularly relevant in studies employing awake animals. Ma et al. (2016) stated in their paper: “Awake mice were imaged head-fixed but positioned on a saucer wheel and were free to run during imaging. The motion of the wheel was monitored throughout imaging using a webcam synchronized with image acquisition. All periods of running were removed, and ‘resting-state’ epochs were defined as periods of at least 30 s of continuous rest.” Over a duration of 30 s or more, a head-fixed awake mouse likely whisks volitionally (Winder et al., 2017). Ma et al. (2016) did not specifically state whether they had taken whisking behavior or fidgeting into account in their definition of the “resting-state.”

Whisking is an important, albeit subtle, behavior in rodents. As pointed out by Sofroniew and Svoboda (2015): “rodents use their mechanosensitive whiskers for a diverse range of tactile behaviors such as navigation, object recognition, and social interactions. These animals move their whiskers in a purposive manner to locations of interest.” Furthermore, whisking triggers cortical dynamics in many brain regions including S1, S2, M1, M2, PPC, thalamus, etc. (O’Connor et al., 2010; Sofroniew et al., 2015; Helmchen et al., 2018). Thus, the individual moments when a mouse volitionally whisks and fidgets should be treated as individual task events; both GCaMP and CBV recordings during these periods should be excluded for analyzing neuronal correlates of resting-state hemodynamic signal. Winder et al. (2017) showed that auditory, whisker stimulation and some volitional whisking events induced distinct hemodynamic responses – an observation appears compelling. Furthermore, Winder et al. (2017) showed that the high correlation between electrophysiological signal (MUA and LFP) was largely driven by these events, a point that warrants further exploration (see section “Technical Limitations”). Additionally, certain transgenic mouse lines are known to have aberrant firing patterns (Steinmetz et al., 2017); it is unknown whether the mouse line used by Ma et al. (2016) had normal basal cortical activity.

The neurophysiological basis of rsFC has been under debate for more than a decade. The debate has been framed in the context of “neurocentric” model. In support of this view, in the spatial domain, converging evidence from animals and human suggests that the interregional correlation patterns derived from electrophysiological recording recapitulate BOLD rsFC (Hacker et al., 2017; Kucyi et al., 2018).

Experiments designed to specifically test the “neurocentric” model only appeared in recent years (Matsui et al., 2011; Ma et al., 2016; Jaime et al., 2017; Mateo et al., 2017; Winder et al., 2017). The two independent studies (Jaime et al., 2017; Winder et al., 2017) employed concurrent electrophysiological recording and hemodynamic measurement, and found weak LFP–BOLD correlation. In particular, the study by Winder et al. (2017) suggests that hemodynamic signal may reflect a combination of potential sources, including behavior, local neural activity, and putatively non-neural processes, calling for careful definition of the “resting-state.” This may be especially relevant in studies using animal models.

## Technical Limitations

The weak correlation between ongoing neuronal activity and hemodynamic signals during the resting-state is somewhat unexpected, and is not in line with the “neurocentric” doctrine of rsfMRI. Several technical issues must be critically reviewed when interpreting this finding. Jaime et al. (2017) performed LFP and rsfMRI recordings in the striatum of anesthetized rats. The anesthetic regime, a combination of low dose isoflurane and dexmedetomdine, has been shown to preserve brain networks (Lu et al., 2012; Brynildsen et al., 2016). But LFP signals recorded with this regime are dominated by low frequency (delta) activity. It is unknown how the use of anesthetics affects neurovascular coupling, particularly in the resting-state. Additionally, the longitudinally implanted microelectrode array unavoidably degrades the quality of rsfMRI signal in voxels around the electrodes. Cytoarchitectonically, inhibitory medium spiny neurons represent 95% of neuronal cells in rat striatum, as opposed to in the neocortex where a majority of neurons are excitatory glutamatergic neurons (Kreitzer and Malenka, 2008). Several studies reported differential neurovascular coupling in the striatum than in the neocortex (Shih et al., 2009, 2012). Specifically, Shih et al. documented bilateral CBV decreases associated with enhanced neuronal activity in the caudate–putamen induced by unilateral noxious electrical stimulation, and that the activation of dopamine D_2_ receptor played a role in this process (Chen et al., 2005). It is thus possible that finding in the striatum may not be generalizable to other brain regions.

The study by Winder et al. (2017) was performed in the neocortex of awake mice, and thus avoided the confounding effects from anesthesia. Nevertheless, they inserted Teflon-coated tungsten-wire stereotrode into the barrel cortex, which necessitated an invasive cranial window preparation, and in some experiments, a cannula was chronically implanted for pharmacological manipulations. Since an acute cranial window preparation likely causes certain degree of tissue insult, it is unknown whether the tissue insult arising from this procedure might have affected neurovascular coupling in the resting-state, although they were able to record robust hemodynamic response to whisker stimulation. In support of this argument, Hudetz et al. (1998) pointed out that spontaneous fluctuation in cerebral blood flow requires the preservation of the flow control system. The fluctuations are absent in focally ischemic cortical territories when the ischemia is severe.

## Potential Roles of Astrocyte in rsfMRI Signal

The potential roles of astrocytes in rsfMRI have been largely ignored. Neurons and associated astrocytes are organized in large-scale synaptic and astrocytic networks. Complex signaling within and between these networks causes fluctuations in cerebral metabolic rate of oxygen (CMRO_2_) and hemodynamic response (Attwell and Iadecola, 2002). The BOLD signal is vascular in origin, and ultimately relies on vasoactive substance release. Neuronal activity is known to cause the release of many vasoactive agents, including H^+^, K^+^, adenosine, arachidonic acid metabolites, nitric oxide (NO) etc. (Iadecola and Nedergaard, 2007). On the other hand, astrocytes are strategically positioned between neuronal and vascular systems: astrocytes forms end-feet on capillaries and arterioles while having contacts with synapses (Mishra, 2017). Importantly, astrocytes are known to express many ion channels, receptors, transporters, and vesicles (Verkhratsky and Nedergaard, 2018). Indeed, astrocytic Ca^2+^ signaling has been shown to play an role in regulating neurovascular coupling (Zonta et al., 2003) and in controlling vessel tone (Rosenegger et al., 2015). Furthermore, several lines of evidence suggest that alterations in parenchymal vascular tone influence astrocytic Ca^2+^ activity and potentially neuronal activity as well, such that the “hemo-neural” hypothesis has been proposed (Moore and Cao, 2008; Kim et al., 2016). Modeling studies suggest that cyclic neuron-astrocyte cross talk could produce slow oscillations in BOLD signal (DiNuzzo et al., 2011; DiNuzzo, 2015). Thus, in addition to the “neurocentral” model, an alternative testable hypothesis could be that the spontaneous BOLD fluctuation is at least partially caused by spontaneous astrocyte activity. Additionally, the role of spontaneous vasomotion in spontaneous BOLD fluctuation may warrant further investigation.

Imaging of astrocytic activity has traditionally relied on fluorescent dyes (Mulligan and MacVicar, 2004; Scemes and Giaume, 2006; Schulz et al., 2012; Otsu et al., 2015). The development of transgenic mouse lines, viral vector targeting strategies and genetically encode Ca^2+^ indicators has made astrocyte-specific recording and manipulation more readily available (Biesecker et al., 2016; Poskanzer and Yuste, 2016; Takata et al., 2018). For example, using transgenic approaches, Takata et al. (2018) generated mice expressing channelrhodopsin specifically in neurons or in astrocytes, and measured BOLD response to astrocyte-specific optogenetic stimulation. They reported that optogenetic activation of astrocytes, in the absence of apparent neuronal modulation, evoked BOLD response. It is thus conceivable that spontaneous activity in astrocyte may cause spontaneous vasodilation and vasoconstriction, leading to variations in BOLD signal that are decoupled from spontaneous neural activity. Future experiments that combine optical readouts of neuronal and astrocytic calcium activities with hemodynamic measurement may shed light on the origins of rsfMRI signal.

## Author Contributions

All authors listed have made a substantial, direct and intellectual contribution to the work, and approved it for publication.

## Conflict of Interest

The authors declare that the research was conducted in the absence of any commercial or financial relationships that could be construed as a potential conflict of interest.
